# *Klebsiella aerogenes* Adhesion Behaviour during Biofilm Formation on Monazite

**DOI:** 10.3390/microorganisms11051331

**Published:** 2023-05-18

**Authors:** Arya Van Alin, Melissa K. Corbett, Homayoun Fathollahzadeh, M. Christian Tjiam, Andrew Putnis, Jacques Eksteen, Anna H. Kaksonen, Elizabeth Watkin

**Affiliations:** 1 Curtin Medical School, Curtin University, Bentley, WA 6102, Australia; a.vanalin@postgrad.curtin.edu.au (A.V.A.); homayoun.fathollahzadeh@curtin.edu.au (H.F.); christian.tjiam@uwa.edu.au (M.C.T.); 2 The Institute for Geoscience Research, School of Earth and Planetary Sciences, Curtin University, Bentley, WA 6102, Australia; 3 Wesfarmers Centre of Vaccines and Infectious Diseases, Telethon Kids Institute, Nedlands, WA 6009, Australia; 4 Centre for Child Health Research, The University of Western Australia, Nedlands, WA 6009, Australia; 5 Institut für Mineralogie, University of Münster, 48149 Münster, Germany; 6 WA School of Mines, Minerals, Energy and Chemical Engineering, Curtin University, Waterford, WA 6152, Australia; jacques.eksteen@curtin.edu.au (J.E.); anna.kaksonen@csiro.au (A.H.K.); 7 CSIRO Environment, Floreat, WA 6014, Australia; 8 School of Science, Edith Cowan University, Joondalup, WA 6027, Australia

**Keywords:** biofilm, *Klebsiella aerogenes*, monazite, extracellular DNA (eDNA), surface attachment

## Abstract

The adsorption behaviour of micro-organisms during the initial attachment stage of biofilm formation affects subsequent stages. The available area for attachment and the chemophysical properties of a surface affect microbial attachment performance. This study focused on the initial attachment behaviour of *Klebsiella aerogenes* on monazite by measuring the ratio of planktonic against sessile subpopulations (P:S ratio), and the potential role of extracellular DNA (eDNA). eDNA production, effects of physicochemical properties of the surface, particle size, total available area for attachment, and the initial inoculation size on the attachment behaviour were tested. *K. aerogenes* attached to monazite immediately after exposure to the ore; however, the P:S ratio significantly (*p* = 0.05) changed in response to the particle size, available area, and inoculation size. Attachment occurred preferentially on larger-sized (~50 µm) particles, and either decreasing the inoculation size or increasing the available area further promoted attachment. Nevertheless, a portion of the inoculated cells always remained in a planktonic state. *K. aerogenes* produced lower eDNA in response to the changed surface chemical properties when monazite was replaced by xenotime. Using pure eDNA to cover the monazite surface significantly (*p* ≤ 0.05) hindered bacterial attachment due to the repulsive interaction between the eDNA layer and bacteria.

## 1. Introduction

In each ecosystem, micro-organisms live in two distinctive subpopulations: free-living or planktonic cells and sessile or biofilm-forming cells [1]. Biofilms are a universal feature of microbial life and, from a human point of view, can be both detrimental and advantageous depending on the circumstances [2]. Biofilms can pose a great health risk by causing more severe symptoms in patients and can lead to significantly higher resistance to antimicrobials [3,4]. In the oil and gas industry, biofilms have been documented as causing biofouling and corrosion. In contrast, biofilms are advantageous for industries benefiting from biotechnological processes such as bioleaching, biofuel cells, bioremediation, and biofertilisers [5]. Prevention or promotion of biofilm formation and development requires detailed understanding of the underlying mechanisms as well as the intrinsic and extrinsic factors affecting the effectiveness of attachment. Adherence to a surface is one of the main capacities of the sessile subpopulation; regardless, attachment to a solid surface is not a requirement to form a biofilm [6,7]. In surface-attached biofilms, only the bottom layer of the cells is directly attached to a substratum [6]. However, in nonsurface biofilm, microbial cells are attached to each other and form flocs of biofilm. Some bacteria, such as *Pseudomonas aeruginosa,* establish chronic infections by forming biofilm flocs [8,9], and in natural environments, marine particles gel and form clumps during cyanobacteria and microalgal blooms [9], which are some examples of nonsurface-attached biofilms. Micro-organisms are capable of attaching to any surface in general, both biotic such as micro-organisms and abiotic such as minerals, with the ability to attach or detach from a surface in response to the environmental conditions [2]. These cycles of attachment and detachment can promote either planktonic or biofilm lifestyle over the other. Each of these populations has a functional role in the ecosystem, and changes in the planktonic to sessile (P:S) ratio in any ecosystem can change the microbial functions in that ecosystem [5,10]. This simple yet significant feature of microbial life can be employed to engineer microbial functions to demote or promote certain activities, such as bioleaching, bioremediation, biodegradation, bioconversion, or antimicrobial resistance.

Bioleaching has attracted enormous interest in the mining industry in recent decades [11], and despite the numerous studies on the significance of planktonic and biofilm subpopulations in bioleaching of sulphide minerals, the translation of this research for promoting or demoting either of these subpopulations to whole processes is still in its infancy. In the bioleaching of rare earth elements (REE) bearing phosphate minerals, very limited data is available regarding the behaviour of either the planktonic or sessile populations within these systems. Previous studies on the bioleaching of monazite [12,13] refer to microbial attachment, and van Alin et al. (2023) is the only study addressing biofilm formation on monazite and xenotime. Fathollahzadeh et al. (2018) reported that the bioleaching rate is higher when micro-organisms are in contact with the mineral surface [12]. Our previous studies have shown that *K. aerogenes* biofilm formation occurs in three characteristic stages [14]. Initial attachment occurred during the first 4–8 h of microbial exposure to the ore surface (1), followed by (2) colonisation of the surface, with a mature biofilm maintained for several days, and (3) finally, cells detached from the ore surface, marking the last stage, biofilm dispersion [14]. Physical imperfections of REE-phosphate ore surfaces enhanced microbial attachment and, as a result, biofilm formation by *Klebsiella aerogenes*. However, in contrast, the mineralogy or chemical composition of the ores’ surface neither promoted nor demoted microbial attachment or biofilm formation [14].

As the initial attachment can be critical in determining the progression to biofilm development, promoting a higher sessile or a planktonic lifestyle [2,5] can strongly influence the bioleaching process. Extrinsic factors, such as the chemophysical condition of a surface, environmental conditions, the size and shape of the available particles, and the total number of the planktonic cells per given space of attachment, can all contribute to the successful attachment and colonisation of a surface [2]. Additionally, intrinsic factors, such as extracellular appendages or the composition of extracellular polymeric substances (EPS) produced by microbes, can change the attachment behaviour [2,15,16]. EPS is known to play a critical role in mediating the initial adhesion of planktonic cells to surfaces, aiding in the stability of the biofilm structure, and providing safety from environmental stresses [15,16,17]. In bioleaching, EPS increases the interface reaction in favour of leaching [18]. EPS also plays another critical role by providing a microenvironment where some of the bioleaching processes, such as dissolution or complexation, occur, hence acting as a micro-biochemical reactor [19,20]. The detailed role of EPS as a microenvironment for bioleaching is described elsewhere [20,21]. In brief, a 50–100 nm space between the outer membrane of micro-organisms and the surface layer of metal-containing ores is filled with EPS. This EPS matrix acts as a biochemical bridge connecting the surface of the substratum and microbes, where micro-organisms release or store the biochemical agents for bioleaching, such as organic/inorganic acids, metal reducing/oxidising enzymes, chemical shuttles capable of reducing or oxidising metals or some other elements, such as sulphur, and bio-chemical shuttles capable of complex formation with the dissolved metals [21,22]. Extracellular DNA (eDNA) is one of the main components of EPS and is involved in many of its functions, such as initial attachment and the integrity and stability of the biofilm structure [17,23,24]. The presence of eDNA can play critical roles in the attachment of micro-organisms to sulphide minerals [25] or metallic surfaces [26] and largely influences microbial behaviour at the surface of the ore [24,27,28]. Whether eDNA promotes the microbial attachment, for example, *Shewanella chilikensis* attachment to steel [26], or demotes it, as in *Caulobacter crescentus* attachment to polystyrene and polyvinylchloride [28], seems to be specific to each type of micro-organism and surface.

In our previous studies on monazite, the changes in pH, phosphate concentration, and released REE were tested using different phosphate solubilizing micro-organisms [13,29], and *K. aerogenes* had the highest phosphate dissolution rate and REE bioleaching performance from monazite [30]. We also explained the role of planktonic and sessile subpopulations of *K. aerogenes* during the bioleaching of monazite [12,31] and biofilm formation [14]. The effect of differences in chemical and physical properties of the monazite ore surface on *K. aerogenes* attachment and biofilm formation has been discussed by van Alin et al. (2023). The current study aims to shed light on the very first stage of *K. aerogenes* biofilm formation on monazite surfaces. The effects of both the physical and chemical properties of the ores’ surfaces on attracting or deflecting either planktonic or sessile populations were studied. Moreover, eDNA production, its role, and the mechanism of action in *K. aerogenes* attachment were studied.

## 2. Material and Methods

### 2.1. Minerals

A high-grade monazite ore (Mt Weld Mine, Laverton, Western Australia) and xenotime beneficiation concentrate (mineral beneficiation is a process by which valuable constituents of an ore are concentrated by means of a physical separation process; the sample was donated by Louis de Klerk, Northern Minerals, WA, Australia) were sterilised by gamma irradiation at 50 kGy for 11 h (ChemCentre, Bentley, Western Australia) to inactivate indigenous micro-organisms. Monazite has high concentrations of light rare earth metals and xenotime has a high concentration of heavy rare earth metals (Tables S1–S3), as determined by X-ray diffraction (XRD), and inductively coupled plasma mass spectrometry (ICP-MS). The main minerals in the high-grade monazite were monazite in the form of (Ce, La, Pr, Nd, Sm)PO_4_ and florencite Al_3_(Ce, La, Nd, Sm, Ca)(PO_4_)_2_(OH)_6_, and main minerals in the xenotime beneficiation concentrate were quarts (SiO_2_) and aluminosilicate minerals (Al_2_SiO_5_), and xenotime (Y, Dy, ER, Yb, Gd)PO_4_.

### 2.2. Micro-Organism and Culture Conditions

All biological experiments were performed using *Klebsiella aerogenes* ATCC 13048. The National Botanical Research Institute’s phosphate medium (NBRIP (g L^−1^): 5 MgCl_2_(H_2_O)_6_, 0.25 MgSO_4_(H_2_O)_7_, 0.2 KCl, 0.1 NH_4_SO_4_, 2 KH_2_PO_4_, 30 glucose, pH 6.2 ± 0.4) was used as the growth medium. The cultures were incubated at 30 °C and 120 rpm.

### 2.3. Cell Enumeration with Flow Cytometry

A flow cytometry assay for enumerating bacteria was used in this study, as previously described [14], with some modifications. Bacterial samples were fixed in 2% glutaraldehyde solution (Sigma, Roedermark, Germany) and assessed in a log-fold dilution series. First 100 µL of the fixed bacterial sample was stained with 1 µL of SYTO 85 (5 mM; Invitrogen, Waltham, MA, USA) for 20 min at room temperature in the dark. Then bacteria were suspended in a final volume of 1 mL of 0.22 µM filter-sterile NBRIP medium and kept on ice in the dark before acquisition on a 4-laser (405 nm, 488 nm, 561 nm and 640 nm) Attune NxT Acoustic Focusing Flow Cytometer (Invitrogen, USA) equipped with side-scatter filters on BL-1 (488 nm) and VL-1 (405 nm) detectors, to aid in small particle discrimination. The cytometer was set to acquire a volume of 50 µL at a flow rate of 25 µL min^−1^ and was set to trigger at a SSC-H threshold value of 0.1 × 10^3^. True bacterial events were discriminated from electronic noise using negative controls that contained NBRIP only or NBRIP and SYTO 85 in the absence of bacteria. A primary gate was used to identify bacterial events through 405 nm and 488 nm SSC profiles, followed by SYTO 85 fluorescence. The concentration of bacteria in each sample was derived by dividing the number of SYTO 85^+^ events by the acquisition volume. Data was exported from the Attune NxT software as FCS3.0 files and analysed in FlowJo v10.7 (BD Biosciences, Franklin Lakes, NJ, USA).

### 2.4. Bacterial and eDNA Attachment Behaviour

Bacterial and eDNA attachment behaviour were studied using monazite and *K. aerogenes* cells and/or DNA.

#### 2.4.1. Effects of the Inoculum Size and Available Surface Area for Attachment on Bacterial Adsorption Behaviour

The bacterial adsorption to the mineral surfaces was studied in terms of the ratio of planktonic cells against sessile (attached) cell subpopulations. The number of the planktonic cells of *K. aerogenes* was determined using flow cytometry at different experimental settings. The number of the attached cells was calculated by deducting the enumerated planktonic cells from the initial inoculum size (1 × 10^7^ cell mL^−1^), and used to calculate the planktonic:sessile population ratio (P:S ratio).

The area available for attachment is dependent on two variables: the initial number of bacteria and the initial available surface area for attachment. The effects of changing any of these two variables on the attachment behaviour were studied (1) using various inoculation sizes (1 × 10^6^, 1 × 10^7^, 5 × 10^7^ cell mL^−1^) with a fixed available area for attachment (1% monazite slurry), and (2) changing the total available area for attachment in terms of monazite slurry percentage (0.5, 1, 2, 5, and 10%) with a fixed inoculation size of 1 × 10^7^ cells mL^−1^. Samples were taken at 1, 10, 30, 60, 120, 180, and 240 min and immediately fixed with glutaraldehyde (final concentration of 2%) and refrigerated until analysis. This experiment was performed in triplicate. The attachment performance of cells on glass surfaces was studied in conical flasks using phosphate-deficient NBRIP and NBRIP amended with dissolved phosphate. Attachment to monazite was tested by adding 1% monazite to the flask using phosphate-free NBRIP medium.

#### 2.4.2. Effects of the Surface Chemical Properties on Bacterial Adsorption Behaviour

DNA was extracted from a 10 mL aliquot of a pure culture of *K. aerogenes* grown in NBRIB media after harvesting cells by centrifugation at 10,000× *g*. The supernatant was discarded, and the cell pellet was used for DNA extraction using DNeasy Kit (Qiagen, Hilden, Germany), following the manufacturer’s procedure. The DNA quantity was measured using Qubit kit (Invitrogen, USA), following the manufacturer’s guidelines. The extracted DNA was used as a pure source of *K. aerogenes* eDNA to evaluate its attachment of eDNA and its impact on bacterial attachment.

The eDNA attachment on monazite surfaces was assessed at concentrations of 5, 25, 50, and 100 ng mL^−1^ in glass flasks containing NBRIP medium in the absence of bacterial cells. The attachment of eDNA to glass flask surfaces was used as the control, also in the absence of bacterial cells. The effects of attached eDNA on *K. aerogenes* adsorption behaviour were studied using monazite ore with and without preconditioning with eDNA. In the absence of bacteria, monazite surface was initially exposed to various concentrations (0, 5, 25, 50 and 100 ng) of eDNA for 60 min. Then, *K. aerogenes* was added to the flasks at an initial cell number of 1 × 10^7^ cell mL^−1^ and the flasks were incubated at 30 °C and 120 rpm for four hours. Samples were taken at 1, 10, 30, 60, 120, 180, and 240 min and immediately fixed with glutaraldehyde (final concentration of 2%) and refrigerated until enumeration of the planktonic population. The experiment was performed in triplicate.

### 2.5. Live Imaging and Evaluation of eDNA Production

To visualise the eDNA production during all three stages of biofilm formation, confocal laser scanning microscopy (CLSM) was performed with live bacteria attached to monazite minerals. Genomic DNA was stained with Hoechst 33342 (AATBio, Sunnyvale, CA, USA) and eDNA was stained with DiTO-1 (AATBio, USA), a highly specific fluorochrome dye that stains DNA external to the cell walls of living cells, i.e., eDNA. Samples were taken from bioleaching experiments conducted in conical flasks (phosphate-free NBRIP medium, 1% slurry of monazite, 10^7^ cells mL^−1^ inoculum size) with monazite grains extracted at 4, 8, 16, 24 h, and 2, 3, 5, 8, 11, 14 days after inoculation. The samples (1 mL) were collected in microtubes and allowed to settle at room temperature for 1 min. The supernatant containing the planktonic subpopulation was discarded, and the solids were gently washed with sterile NBRIP medium to remove any remaining planktonic or loosely attached cells from the minerals. Samples were stained with the two fluorochromes (1 µL mL^−1^) for 20 min and then, transferred to a µ-Slide 2 Well Glass Bottom microscope slide (ibidi, Gräfelfing, Germany). CLSM images were collected in Z-stack mode with 3.5–7 micron intervals (×10 and ×20 objectives) or ≤1 micron intervals (×40 and ×100 objectives). The images are shown in Maximum Intensity Projections mode or Maximum Intensity Projections in 3D. To further visualise eDNA production, the acquired Z-stack CLSM images were reanalysed in IMARIS v 9.7 (Oxford Instruments, Oxford, UK). The surface of monazite samples was reconstructed using the “Surface” tool, and the cells were modelled using “Spots” tool following IMARIS v 9.7 user guidelines to provide a better 3D view of both cells and the surface.

### 2.6. Fluorescent Microscopy

In order to study bacterial behaviour during the first minutes (1–10 min) of *K. aerogenes* contact with high-grade monazite ore, images from bacterial attachment were recorded using Olympus BX51 upright microscope. Prestained cells with Hoechst 33342 (1 µL mL^−1^, 20 min) were added to 10 mg of each mineral sample in microtubes at 10^7^ cells mL^−1^, gently mixed, and immediately studied using WB: blue excitation (wide band) filter (Ex 465/15, FT 500, LP 515).

### 2.7. Measurement of Surface Electrical Potential

A ZS ZEN3600 Zetasizer (Dispersion Technology Software v5.10, 2008, Malvern, UK) and low-volume plastic disposable cuvettes (ZEN112, Malvern, UK) were used to measure the surface electrical potential [32].

### 2.8. Statistics

Data preparation for statistical analysis was conducted in Microsoft Excel (2016) and GraphPad Prism v.9. Two-way ANOVA was used for comparison, and a *p*-value < 0.05 was considered significant. The statistical analysis, including calculated *p*-values, is provided as Supplementary Material.

## 3. Results

### 3.1. Attachment Behaviour in Response to the Monazite Particle Size

**Figure 1:**
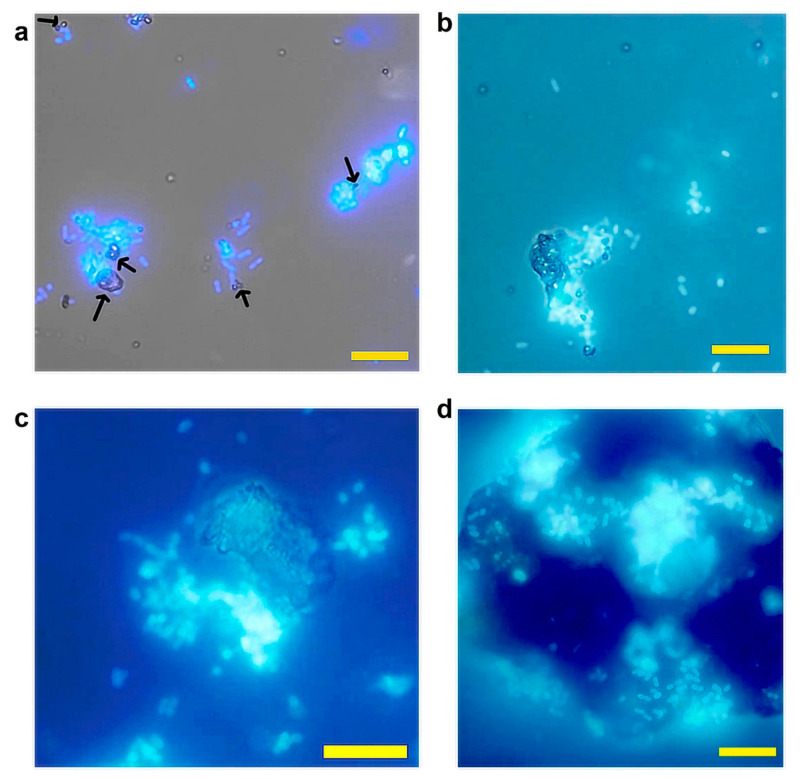
Epifluorescence microscope images of *Klebsiella aerogenes* on high grade monazite ore over the first 10 min after inoculation. (**a**) Bacterial cells gathering around small particles of approximately ≤10 µm, (**b**,**c**) partially attached and partially gathered around larger ore particles of ~10–20 µm (**d**) and bacterial cells mainly attached to the surface of larger particles (≥50 µm). Black arrows point to the small particles. Scale bar 20 µm.

*K. aerogenes* attachment to monazite occurred immediately after adding the microbial culture to the system (Figure 1). Depending on the particle size, the microbial behaviour was different. *K. aerogenes* cells were mainly gathered around small (approximately ≤10 µm) and mid-size (~20 µm) monazite particles while they were attached to the surface of larger monazite particles (~50 µm) (Figure 1).

### 3.2. Cell Attachment Behaviour in Response to the Nutrient Amendment and Surface Material Available for Attachment

**Figure 2:**
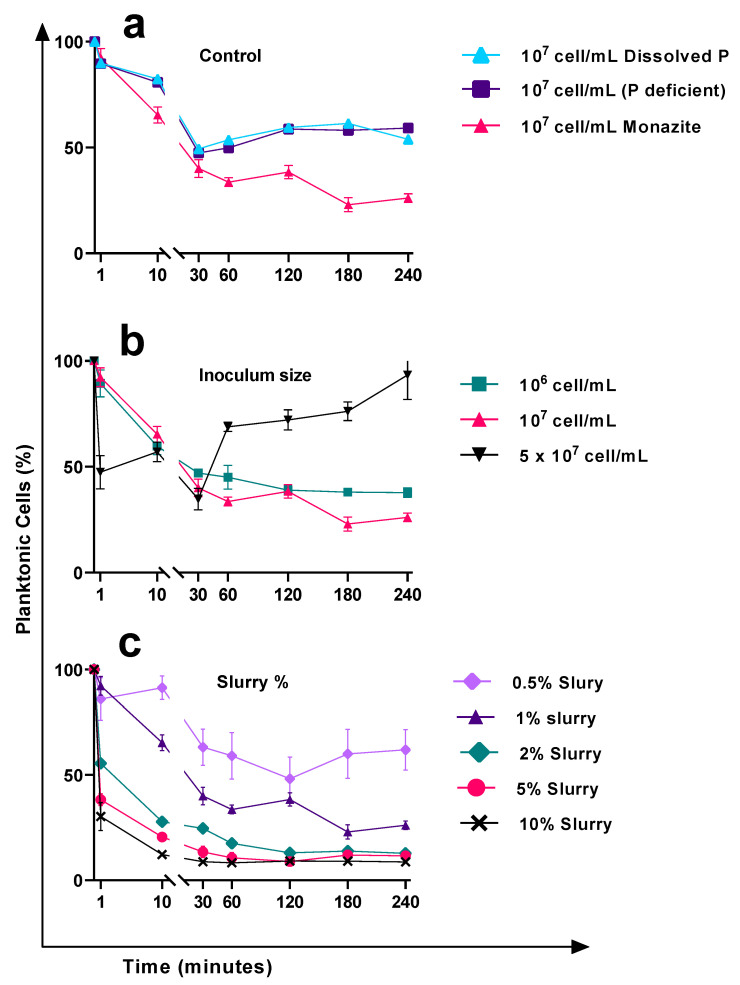
The percentage of planktonic *Klebsiella aerogenes* during the early attachment phase of biofilm formation on glass and high-grade monazite ore. (**a**) Attachment to glass surface in NBRIP medium at an initial inoculum size of 1 × 10^7^ cell mL^−1^, with (dissolved P) or without (P deficient) phosphate in the medium, and to both glass and monazite using 1% slurry monazite as the only source of phosphate and (**b**) with varying initial inoculum sizes with 1% slurry of monazite, and (**c**) with varying amounts of monazite at a fixed initial inoculum size of 1 × 10^7^ cell mL^−1^. The depicted data are bacterial cells enumerated by flow cytometry.

In control samples (no monazite), glass was provided as the only available surface for attachment. The absence (phosphate (PO_4_) deficient control) or presence (dissolved PO_4_ control) of phosphate did not cause a significant change in the attachment of the bacteria on glass with an inoculation size of 1 × 10^7^ cell mL^−1^ (*p* ≥ 0.05, Figure 2a). Moreover, there were no significant changes in the cell number (*p* ≥ 0.05), signifying no or very slow reproduction rate for the *K. aerogenes* during the first 4 h of inoculation. Bacteria rapidly and consistently attached themselves to the glass over the first 30 min. The P:S ratio of the two control samples decreased from 9:1 (~10% attachment efficiency) in the first minute to approximately 1:1 (~50% attachment efficiency) after 30 min. Then, the attachment efficiency gradually decreased to 40% (P:S ratio of 1.5:1) by two hours and remained relatively stable until the end of the experiment (4 h). In the presence of monazite (1% slurry, 1 × 10^7^ cell mL^−1^), the attachment efficiency was relatively higher than the glass controls and improved to ~60% of the initial inoculated cells (a P:S ratio of ~1:1.5) after 30 min, compared to ~50% efficiency of the control samples. The planktonic subpopulation continued to decrease to ~25% of the initial inoculum size (a P:S ratio of ~1:3) by the end of the experiment (Figure 2a).

### 3.3. Cell Attachment Behaviour in Response to the Changes in the Initial Inoculum Size

In the presence of a fixed amount of monazite (1% slurry), varying the cell numbers significantly changed the efficiency of the attachment (*p* ≤ 0.05) (Figure 2b). The attachment behaviour of *K. aerogenes* to monazite at a lower inoculum size of 1 × 10^6^ cell mL^−1^ was fairly similar to that of the 1 × 10^7^ cell mL^−1^, but at a slightly lower efficiency. At the very first minute of exposure to monazite, the number of attached cells with an initial 1 × 10^6^ cell mL^−1^ was similar to when 1 × 10^7^ cell mL^−1^ was used (P:S ration 0f 9:1). The attachment efficacy improved over time, from approximately 10% to 60%. In contrast, increasing the inoculum size to 5 × 10^7^ cell mL^−1^ changed the attachment behaviour of *K. aerogenes*. The planktonic subpopulation decreased to half within the very first minute of exposure to monazite, resulting in a P:S ratio of 1:1 compared to 9:1 (10% attachment ratio) for the other samples (Figure 2b, black line). The attachment efficiency fluctuated to ~40% at 10 min and then 60% after 30 min. However, the attachment behaviour of the bacteria changed after this time point. Two hours after exposure to monazite, the attachment efficacy of both 1 × 10^6^ and 1 × 10^7^ cell mL^−1^ inoculum sizes improved to ~60%, an increased P:S ratio of 1:1.5 (Figure 2b, red and green lines). It remained unchanged to the end of the experiment for the lower inoculum size, while it continued to improve for the initial inoculum size of 1 × 10^7^ cell mL^−1^ (75% attachment efficiency, an enhanced P:S ratio of 1:3 after 4 h). In contrast to these two groups, the attachment behaviour of *K. aerogenes* at a higher initial inoculum size of 5 × 10^7^ cell mL^−1^ drastically changed after 30 min. The attachment behaviour reversed and largely decreased from ~66% (a P:S ratio of 1:2), to ~25% (a P:S ratio of 3:1) at 60 min. This sharp drop continued to ~7% (a P:S ratio of 13:1) by the end of the experiment.

### 3.4. The Effects of the Available Area for Attachment on the Attachment Efficiency

The inoculum size is not the only influencing factor changing the attachment efficiency. In the presence of monazite, at a constant inoculum size of 1 × 10^7^ cells mL^−1^, varying the available area for attachment in terms of changing the slurry percentage of monazite resulted in different attachment behaviours (Figure 2c). While the general pattern was a continuous decrease in the planktonic subpopulation, the attachment efficiency was significantly different (*p* ≤ 0.05). Attachment behaviour of the bacteria using higher amounts of monazite (2, 5, and 10%, slurry, Figure 2c) was fairly similar; sharp attachment efficiency was observed during the very first minute of exposure to monazite, which was significantly higher than what was observed for 1% slurry (*p* ≤ 0.05). The attachment efficiency of samples with ≥1% monazite slurry significantly improved over time (*p* ≤ 0.05) and stabilised at a P:S ratio of ~1:3, approximately 75% attachment efficiency 3 h after inoculation. Increasing the slurry to 2, 5, and 10 percent significantly improved the attachment efficiency in terms of both attachment efficiency (P:S ratio) and time (*p* ≤ 0.05). For 2% and 5% slurry, approximately 87% and 89% attachment efficiency were achieved after 2 h, and using 10% slurry, 91% attachment efficiency was recorded just an hour after inoculation. In contrast, halving the available monazite (0.5% slurry), resulted in lower attachment efficiency with gradual improvement to a P:S ratio of 1:1 (50% attachment efficiency) two hours after inoculation, followed by a reversal of the attachment behaviour in favour of the planktonic subpopulation, where the attachment efficiency dropped to 40% by the end of the experiment.

### 3.5. eDNA Production by K. aerogenes

**Figure 3:**
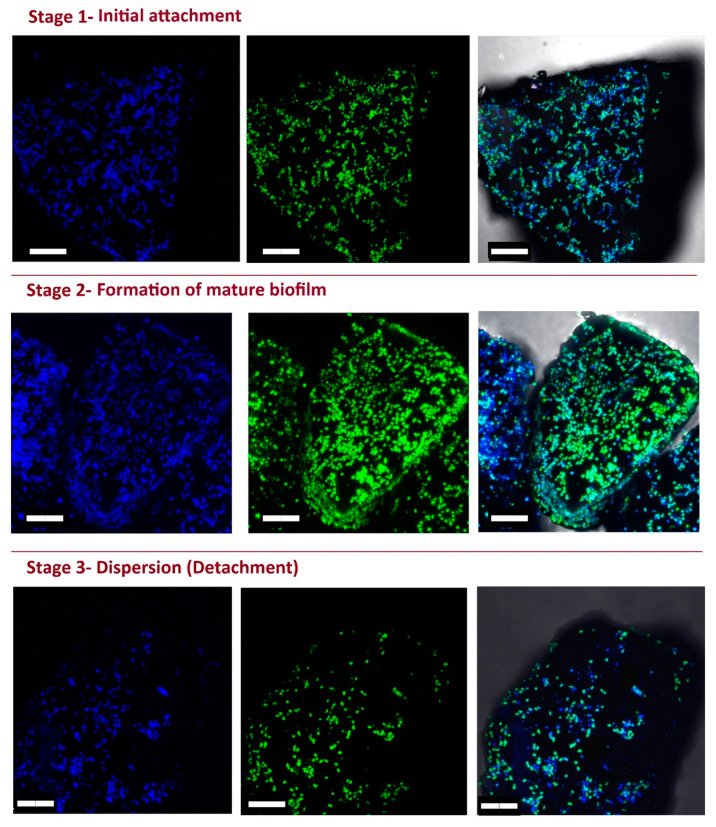
Confocal laser scanning microscopy (CLSM) images of *Klebsiella aerogenes* on the surface of high-grade monazite ore show eDNA (green fluorescent dye) presence at the three stages of biofilm development. Scale bar = 10 µm.

**Figure 4:**
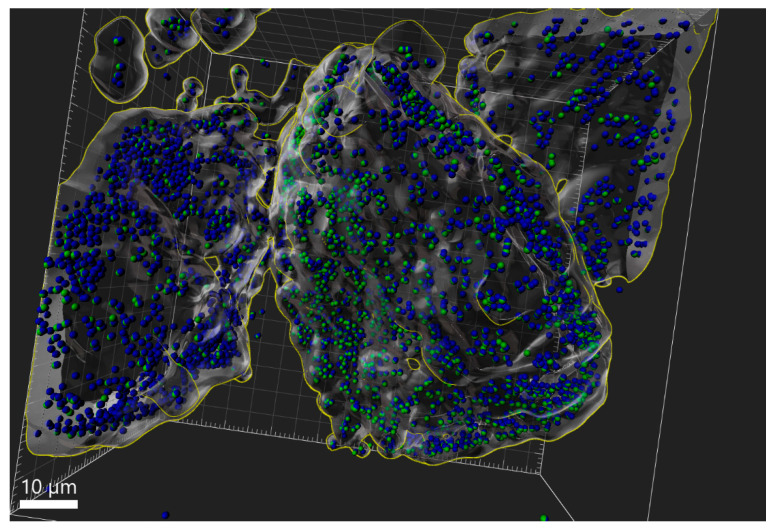
Three-dimensional model of *Klebsiella aerogenes* attachment to the surface of high-grade monazite ore reconstructed using CLSM Z-stack images in IMARIS software (v4). Genomic DNA identification was obtained using Hoechst 33324. In the reconstructed 3D models, cells that were positive for Hoechst 33324 were depicted as a blue sphere. eDNA production was studied using DiTO-1, an eDNA-specific stain (shown in green). Each sphere represents a single bacterial cell. Cells with a positive signal for eDNA were depicted as blue-green spheres against blue-only spheres, which represent no eDNA production.

**Figure 5:**
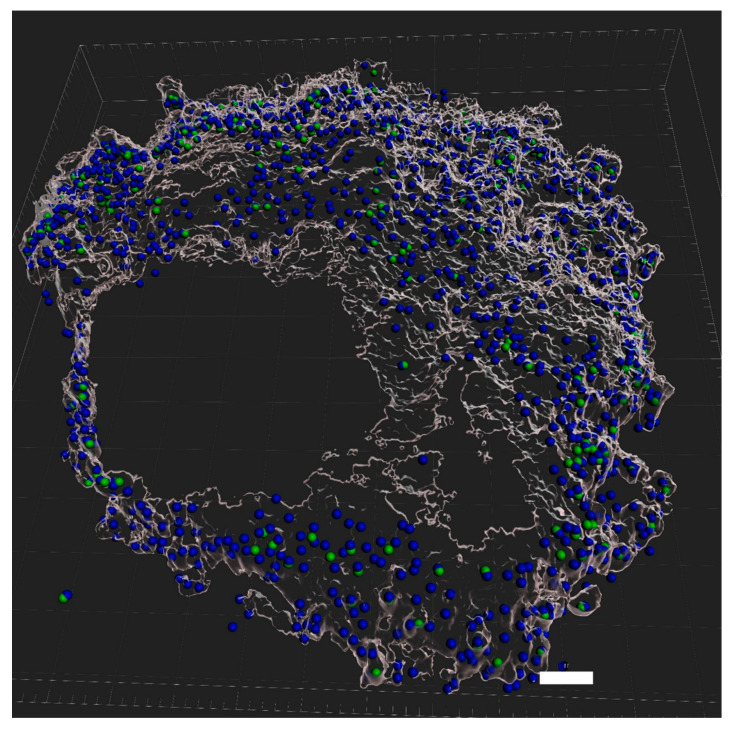
The 3D model of *Klebsiella aerogenes* attachment to xenotime surface reconstructed in IMARIS software (v 9.7). Scale bar: 10 µM. For info on green and blue spheres, please see the caption for Figure 4.

The CLSM live imaging of *K. aerogenes* biofilm demonstrates eDNA production in all three stages of biofilm development (Figure 3); however, not all cells were involved in the production of eDNA. Moreover, the reconstructed models showed that eDNA production differed between different mineral grains. A major proportion of cells attached to some of the mineral grains produced eDNA (blue-green spheres), while for the other grains, very limited eDNA production was detected (blue-only spheres, Figure 4). Therefore, different mineralogy could explain the different patterns of eDNA production. Since CLSM is not capable of determining the mineral composition of different grains in the sample, another phosphate mineral (xenotime) was used to test this hypothesis. The CLSM images clearly showed that eDNA production by *K. aerogenes* cells was different on xenotime (Figure 5). The bacteria produced much less eDNA on xenotime compared to high-grade monazite.

### 3.6. Role of eDNA in Early Attachment and Interaction with the Surface

**Figure 6:**
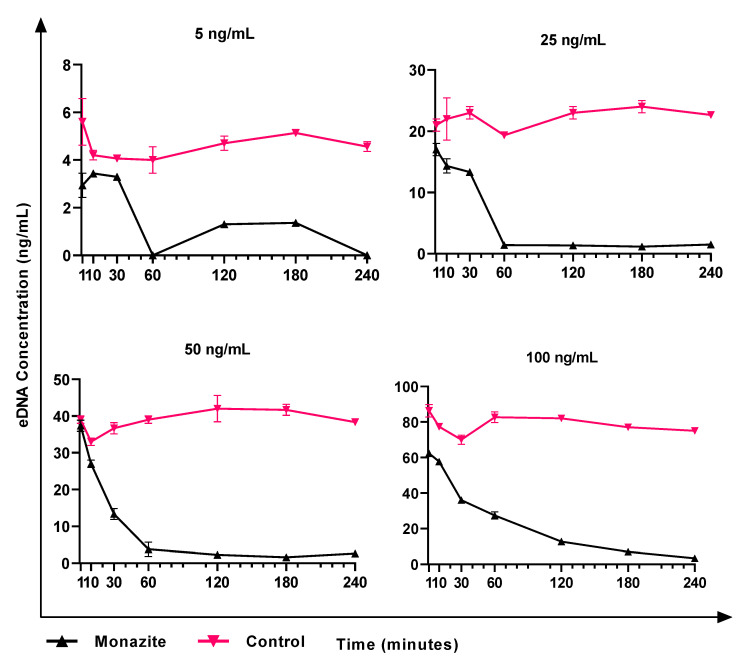
The adsorption of pure eDNA of *Klebsiella aerogenes* to glass (marked as control), and monazite and glass (marked as monazite), in the absence of microbial cells. The attachment efficiency is represented by the eDNA concentration remaining in NBRIP medium.

The eDNA interaction with the surface of minerals was tested using DNA extracted from *K. aerogenes*. In the control sample, where no bacteria were present, the DNA extracted from *K. aerogenes* adhered to the glass surface (Figure 6). By adding monazite to the system, significantly higher attachment was recorded for all tested eDNA concentrations (5–100 ng mL^−1^). eDNA attachment to the glass surface followed a relatively similar pattern (Figure 6), with an improved attachment rate to the glass surface in the beginning followed by a gradual detachment of the adsorbed eDNA, reaching a relatively stable value. In contrast, in the presence of 1% monazite slurry, the general pattern of eDNA adsorption to the surface was a sharp adsorption rate to the surface over the first 60 min of exposure, followed by continuous attachment until a major proportion (~90–95%) of eDNA was attached to the surface. Using 100 ng mL^−1^ eDNA, the eDNA progressively adsorbed to the surface and reached 97% efficiency by the end of the experiment.

### 3.7. Effects of the Surface Preconditioning with eDNA on Attachment of K. aerogenes Cells

**Figure 7:**
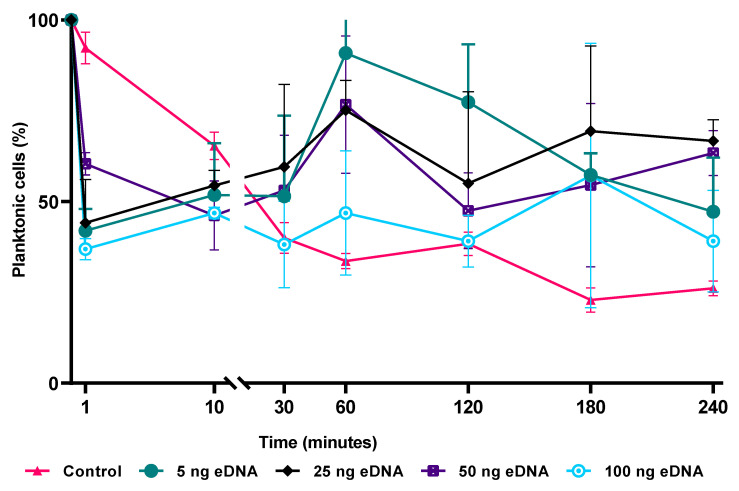
The effect of surface preconditioning with eDNA on the early attachment of *Klebsiella aerogenes* to monazite. The experiment was conducted in NBRIP media with 1% slurry of high-grade monazite ore. Bacterial cells were enumerated using flow cytometry.

To understand the role of eDNA in *K. aerogenes* attachment to the surface, monazite grains were preconditioned with eDNA prior to bacterial inoculation. The pretreatment of the monazite surface with eDNA drastically changed the attachment efficiency of *K. aerogenes* (Figure 7). In the control sample (absence of eDNA at an initial cell concentration of 1 × 10^7^ cell mL^−1^ and 1% slurry monazite), cells continually attached to the surface over the four hours of the test period. During the first 10 min of inoculation, regardless of the amount of eDNA used for preconditioning, all of the preconditioned samples showed a higher attachment of *K. aerogenes* compared to the control flask. In the control flask, *K. aerogenes* attachment efficiency increased steadily over the course of the experiment. However, looking at the general pattern, the preconditioning of the surface with eDNA reduced the attachment efficiency from 30 min onward (Figure 7, green, black, blue, and purple lines). Preconditioning showed an inhibitory effect on the attachment efficiency, with a noticeable difference in the measured cell numbers between the three biological replicas at each time point and inconsistent attachment efficiency between different time points. This signifies a larger adsorption/detachment dynamic in preconditioned samples, therefore resulting in significant fluctuations in attachment efficacy within and between each sampling time point.

## 4. Discussion

In our previous study, it was shown that *K. aerogenes* biofilm formation on phosphate minerals (monazite and xenotime) occurred in three distinctive stages [14]. The formation of mature biofilm has biotechnological advantages in industries such as bioleaching. As attachment to the surface and biofilm formation are the most important prerequisites of contact bioleaching [12], it is, therefore, important to understand this process to further improve and advance bioleaching systems.

The attachment efficiency of *K. aerogenes* to monazite ore surfaces was affected by several factors such as particle size, eDNA attachment, initial cell number, and total available area for attachment (Figure 1, Figure 2 and Figure 7), as has been seen in other systems [2,33]. In general, extrinsic factors, such as nutrient availability in the liquid environment, particle size, which can directly affect nutrient accessibility from an ore, flow and shear forces, pH, ionic strength, physical properties of the surface such as topological imperfections, and chemical properties or mineralogy of the surface, play a role in microbial attachment.

In this study, *K. aerogenes* attachment to monazite occurred immediately after inoculation. However, the attachment behaviour was different in response to the particle size of monazite. For larger particles of monazite (≥50 µm) a notable number of bacteria were attached to the surface over the first minutes of microbial inoculation, whereas for medium-sized (~10–20 µm) and particularly small particles (≤10 µm) bacteria preferred to gather around the particles (Figure 1). Due to their small size, the microscopic particles offer a rapid dissolution rate compared to larger ore particles, thus providing easier access to the nutrient content of the ore, such as phosphate and trace elements [33].

During the early attachment stage of the biofilm formation, a greater proportion of cells remained in the planktonic state in the absence of monazite, either with or without soluble phosphate, as compared to that observed in the presence of monazite without phosphate amendment. The adsorption-detachment dynamics of the *K. aerogenes* to and from the glass surface were maintained significantly (*p* ≤ 0.05) in favour of the planktonic subpopulation (40% attachment) with and without phosphate in the media. In contrast, the attachment efficiency of *K. aerogenes* was significantly (*p* ≤ 0.05) enhanced when cells were exposed to monazite, and by the end of the experiment, the attachment/detachment dynamic stabilised in favour of the sessile subpopulation (75% attachment). Therefore, the surface monazite significantly promoted the sessile subpopulation of *K. aerogenes* compared to a glass surface (*p* ≤ 0.05). Adding monazite to the system provided the cells with a source of phosphate, which could attract bacterial cells to the surface through chemotaxis [2]. In addition, higher attachment efficiency could be due to the nature of the physical surface properties of the glass and monazite. A glass surface provides a fairly smooth interface, while the surface of the high-grade monazite ore is rough and rich in imperfections (Figure S1). The attachment and colonisation of *K. aerogenes* [14] were more favourable in the presence of surface physical imperfections, such as cracks or grooves. Similar results have been reported for *Acidithiobacillus ferrooxidans* attachment to pyrite [19]. Therefore, a surface with more physical irregularities can attract more planktonic cells to attach to and colonise it.

In addition to the physical or chemical quality of a surface, the total available area on the surface altered the attachment efficiency and behaviour of *K. aerogenes*. A planktonic lifestyle is favoured by a higher cell number or a smaller available area. By halving the available space for attachment (decrease from 1% to 0.5% slurry), a lower attachment efficiency was observed. However, using more monazite (2, 5, and 10% slurry) in the system led to a significantly higher (*p* ≤ 0.05) rate of attachment in a shorter time. Moreover, altering the rate of inoculation changed the attachment behaviour when the available area remained unchanged. By varying cell numbers, significant (*p* ≤ 0.05) changes in the efficiency and/or the attachment behaviour of *K. aerogenes* were recorded. Although, by lowering the inoculum size, the attachment efficiency decreased, the general pattern was that of promoting higher attachment over time. In contrast, using higher cell density reversed the attachment behaviour in favour of a planktonic lifestyle.

Regardless of the initial cell numbers or the available area for attachment, the planktonic and sessile subpopulations will reach equilibrium. At a lower available area or a higher cell density (inoculum size), the equilibrium of planktonic vs. sessile subpopulations shifted towards higher attachment, whereas using a smaller initial inoculum size or providing a higher available area for attachment and colonisation shifted the equilibrium towards the sessile lifestyle. Nevertheless, a portion of the cells remained in the planktonic state even in a very high available area for attachment (10% monazite slurry). In brief, factors such as competition for space, nutrients, and safety from environmental stresses contribute to the switch from a planktonic lifestyle to a sessile lifestyle and vice versa. For bioleaching activities, biofilm formation (i.e., sessile lifestyle) is more favourable, as it results in higher dissolution rates of rare earths from monazite ore [12]. Therefore, changes in the P:S ratio and resulting attachment behaviours affect the microbial stability and bioleaching performance. While a higher attachment efficiency can result in timely and optimal biofilm formation, it has been demonstrated that a sharp decrease in planktonic cell number or removal of this population can significantly (*p* ≤ 0.05) change the bioleaching efficiency [34]. Therefore, considering the higher efficiencies reported for contact bioleaching of monazite using *K. aerogenes* [12], this case study indicates that using relatively larger particle sizes with rough surfaces, a higher slurry concentration, or an optimised microbial inoculum size to prompt biofilm formation, promotes contact leaching.

In addition to the chemo-physical conditions of the surface and the environmental conditions, intrinsic microbial properties such as their extracellular appendages and the composition of their EPS can affect attachment efficiency [2,6,35]. The CLSM images (Figure 3) clearly showed that *K. aerogenes* cells produced a significant amount of extracellular DNA, with production detected in all three stages of biofilm formation. Further examination of the eDNA production using a reconstructed 3D model (Figure 4) of the ore grains demonstrated that while the monazite particle in the middle harboured a population of *K. aerogenes* with the majority of the cells producing eDNA, only a small proportion of the attached cells on the other grains produced eDNA. Therefore, eDNA production can vary between individual grains, which could be due to differences in the chemical or mineralogical composition of a mineral surface. To explore this hypothesis, *K. aerogenes* biofilm formation was examined on xenotime. Lower amounts of eDNA were detected in the biofilm of *K. aerogenes* on xenotime, with fewer bacterial cells producing eDNA (Figure 5) in comparison to that produced on monazite (Figure 4). It has been demonstrated that the chemical composition of the mineral surface can affect the attachment behaviour of micro-organisms. *A. ferrooxidans* showed lower attachment affinity to pyrite when grown on elemental sulphur [19]. However, our previous study demonstrated that, *K. aerogenes* showed no selectivity in attachment based on chemical composition or mineralogy on the surface [12,14]. This is in contrast to the current study, which demonstrated that surface mineralogy changed the composition of *K. aerogenes* EPS (Figure 4 and Figure 5) by producing differing amounts of eDNA when grown on xenotime and monazite. According to the observed general patterns based on the CLSM study (Figure S2), when *K. aerogenes* was exposed to light rare earth metals, such as lanthanum, cerium, neodymium, and samarium in monazite ore, it produced a high amount of eDNA. However, using Xenotime ore, which contains heavy rare earth metals, such as dysprosium, yttrium, and ytterbium, exposed this bacterium to a different chemical condition and resulted in remarkably lower eDNA production.

Furthermore, as eDNA is known to play a critical role in initiating or mediating the attachment of planktonic cells to a surface [17], the cell-surface interactions (both attractive and repulsive) were examined as a way to explain the changes in bioleaching efficiencies. Extracted DNA of *K. aerogenes* was used as the eDNA source, and in the absence of bacteria, the eDNA partially attached to the glass; however, it was almost fully attached to the high-grade monazite ore surface at all of the tested concentrations. This demonstrates that eDNA can facilitate *K. aerogenes* attachment to the surface, as it has been shown for some other micro-organisms such as *Pseudomonas, Sulfobacillus,* and *Shewanella* [17,25,26]. Tuck et al. (2022) suggested that preconditioning a metal (steel) surface with eDNA layer promoted *Shewanella chilikensis* attachment; however, preconditioning of a monazite surface with an eDNA layer hindered *K. aerogenes* attachment (Figure 7). The effect of surface preconditioning with an eDNA on the early attachment of *Klebsiella aerogenes* to monazite. The experiment was conducted in NBRIP media with a 1% slurry of high-grade monazite ore. Bacterial cells were enumerated using flow cytometry. The measured electrical potential of each particle (monazite, *K. aerogenes*, and preconditioned monazite with eDNA) showed that the preconditioning of monazite with the eDNA layer resulted in a more negatively charged surface, from −10 mV to −15 mV. On the other hand, *K. aerogenes* has a very negative surface charge (measured at −15 mV). Therefore, bacterial cells were constantly repulsed from the surface of the eDNA-preconditioned monazite (Figure S3) and were incapable of retaining a strong attachment. High variation was observed between the 3 biological replicates of each sample at each time point (Figure 7). Therefore, although Figure 6 clearly shows that eDNA facilitates *K. aerogenes* attachment to the monazite surface, releasing eDNA in a liquid environment to form an eDNA layer on the surface is not a valid mechanism of action. In contrast to Tuck et al. (2022), who showed that released eDNA by *Shewanella* assisted attachment of this micro-organism to the surface by forming an eDNA layer on the surface, forming such a layer by *K. aerogenes* resulted in repulsion of the cells from the monazite surface. Hence, another mechanism involving eDNA allows for the initial attachment. The most potential mechanism could be the production of eDNA alongside other extracellular polymers, such as polysaccharides and proteins, which assists *K. aerogenes* adherence to the surface [17,20,25].

## 5. Conclusions

The initial attachment stage of *K. aerogenes* to the monazite surface can affect colonisation of the surface. Larger particle sizes with more physical imperfections attract more planktonic cells to the surface of monazite compared to small particles. The attachment behaviour analysis examined in terms of the dynamics of planktonic and sessile equilibrium showed a higher attachment to monazite compared to the control glass surface, in which lowering the initial cell concentration shifted the equilibrium of the two subpopulations towards a sessile lifestyle, as did increasing the total available area. eDNA is an important part of the EPS of the *Klebsiella aerogenes* biofilm matrix. While no difference was recorded in the eDNA production of *K. aerogenes* in response to physical properties of the surface, such as physical imperfections, it responded to changes in the chemical properties of the surface (surface mineralogy). *K. aerogenes* produced more eDNA on monazite, which contains light rare earth metals, compared to that produced when exposed to the heavy rare earth content of Xenotime. Analysing eDNA adherence to glass and monazite showed a significantly improved attachment to monazite, implying that eDNA can assist bacterial attachment to the surface during the initial attachment stage of biofilm formation on monazite. Preconditioning the monazite surface with pure eDNA extracted from *K. aerogenes* repulsed bacterial attachment to the surface, suggesting that eDNA assists bacterial attachment as part of the EPS but not by forming an eDNA layer masking the mineral surface.

## Data Availability

The authors confirm that the data supporting the findings of this study are available within the article’s Supplementary Materials or from the corresponding author [E.W.] on request.

## Supplementary Materials

The following supporting information can be downloaded at: https://www.mdpi.com/article/10.3390/microorganisms11051331/s1, Figure S1: Scanning electron microscopy (SEM) image of the smooth surface of glass (a) vs. the rough surface of the high-grade monazite ore (b); Figure S2: The general pattern of eDNA production on monazite (a) and xenotime (b) based on confocal laser scanning microscopy (CLSM). The green fluorescent represents eDNA (eDNA) and blue represent genomic DNA (gDNA). More eDNA was produced using monazite. Yellow circle marks the xenotime grain used for 3D re-construction in Figure 5; Figure S3: The interaction of *Klebsiella aerogenes* cells with surface pre-conditioned with Edna. Table S1: the XRD and phase identification of the high-grade monazite ore conducted by John de Laeter Centre, Curtin University. The COD ID refers to the phase’s identification number in the COD database (http://www.crystallography.net/, access date: 28 September 2021); Table S2: The inductively coupled plasma mass spectrometry (ICP-MS) analysis of high-grade monazite ore conducted by Bureau Veritas, Perth, Australia; Table S3: The XRF (X-ray fluorescence) and ICP-MS composition of xenotime beneficiation concentrate (reported by the provider, Northern Minerals); Statistical data.
