# Supplementary material for: Klebsiella aerogenes Adhesion Behaviour during Biofilm Formation on Monazite
Source: Microorganisms. 2023 May 18;11(5):1331. doi: 10.3390/microorganisms11051331 (PMC10222597; doi:10.3390/microorganisms11051331)
Supplement: Supplementary file 1 [file microorganisms-11-01331-s001.zip › microorganisms-2331644-supplementary.pdf]

Supplementary Material

Klebsiella aerogenes Adhesion Behaviour during Biofilm Formation on Monazite

Arya Van Alin [1,2], Melissa K. Corbett [1,2], Homayoun Fathollahzadeh [1,2], M. Christian Tjiam [1,3,4], Andrew Putnis [2,5], Jacques Eksteen [6], Anna H. Kaksonen [6,7] and Elizabeth Watkin [1,2,8,*]

[1] Curtin Medical School, Curtin University, Bentley, WA 6102, Australia; a.vanalin@postgrad.curtin.edu.au (A.V.A.); homayoun.fathollahzadeh@curtin.edu.au (H.F.); christian.tjiam@uwa.edu.au (M.C.T.)
[2] The Institute for Geoscience Research, School of Earth and Planetary Sciences, Curtin University, Bentley, WA 6102, Australia
[3] Wesfarmers Centre of Vaccines and Infectious Diseases, Telethon Kids Institute, Nedlands, WA 6009, Australia
[4] Centre for Child Health Research, The University of Western Australia, Nedlands, WA 6009, Australia
[5] Institut für Mineralogie, University of Münster, 48149 Münster, Germany
[6] WA School of Mines, Minerals, Energy and Chemical Engineering, Curtin University, Waterford, WA 6152, Australia; jacques.eksteen@curtin.edu.au (J.E.); anna.kaksonen@csiro.au (A.H.K.)
[7] CSIRO Environment, Floreat, WA 6014, Australia
[8] School of Science, Edith Cowan University, Joondalup, WA 6027, Australia
* Correspondence: e.watkin@ecu.edu.au

Table S1. The XRD and phase identification of the high-grade monazite ore conducted by John de Laeter Centre, Curtin University. The COD ID refers to the phase's identification number in the COD database (http://www.crystallography.net/).

Phase	Nominal elemental composition
**Monazite, Ce**	$CePO_4$
**Monazite, La**	$LaPO_4$
**Quartz**	SiO_2
**Goethite**	FeOOH
**Florencite-Ce**	$Al_3(Ce,La,Nd,Sm,Ca)(PO_4)_2(OH)_6$

Table S2. The inductively coupled plasma mass spectrometry (ICP-MS) analysis of high-grade monazite ore conducted by Bureau Veritas, Perth, Australia.

Elements (%)	
**Al**	3.9
**Ca**	1.8
**Fe**	1
**K**	<0.01
**Mg**	0.1
**Mn**	0.06
**Na**	0.1
**P**	8.5
**Si**	1.6
**Ti**	0.4
**Y**	0.18
**La**	11
**Ce**	15
**Pr**	2.1
**Nd**	7.2
**Sm**	0.97
**S**	0

Table S3. The XRF (X-ray fluorescence) and ICP-MS composition of xenotime beneficiation concentrate (reported by the provider, Northern Minerals).

Compound	XRF analysis (Mass %)	ICPMS analysis
**SiO_2**	52.5	Not measured
**Al_2O_3**	6.1	7.82
**Y_2O_3**	8.6	5.23
**Dy_2O_3**	1	1.03
**Er_2O_3**	0.7	0.63
**Yb_2O_3**	0.5	0.53
**Gd_2O_3**	0.4	0.47
**Sm_2O_3**	0.2	0.21
**Fe_2O_3**	9.1	11.94
**CaO**	0.255	1.38

**U_3O_8**	0.055	0.05
**P_2O_3**	Not measured	12.77

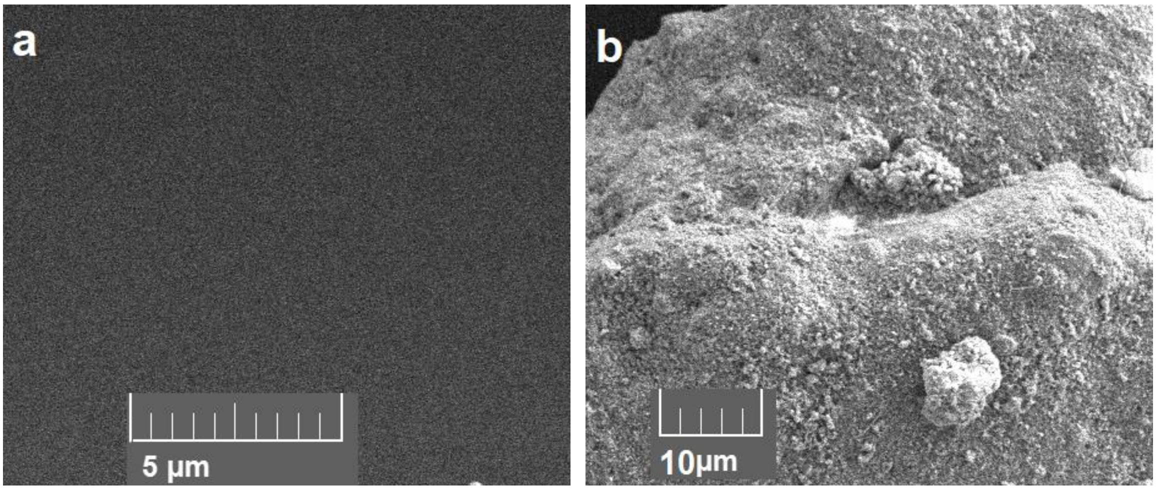

Figure S1. Scanning electron microscopy (SEM) image of the smooth surface of glass (**a**) vs. the rough surface of the high-grade monazite ore (**b**).

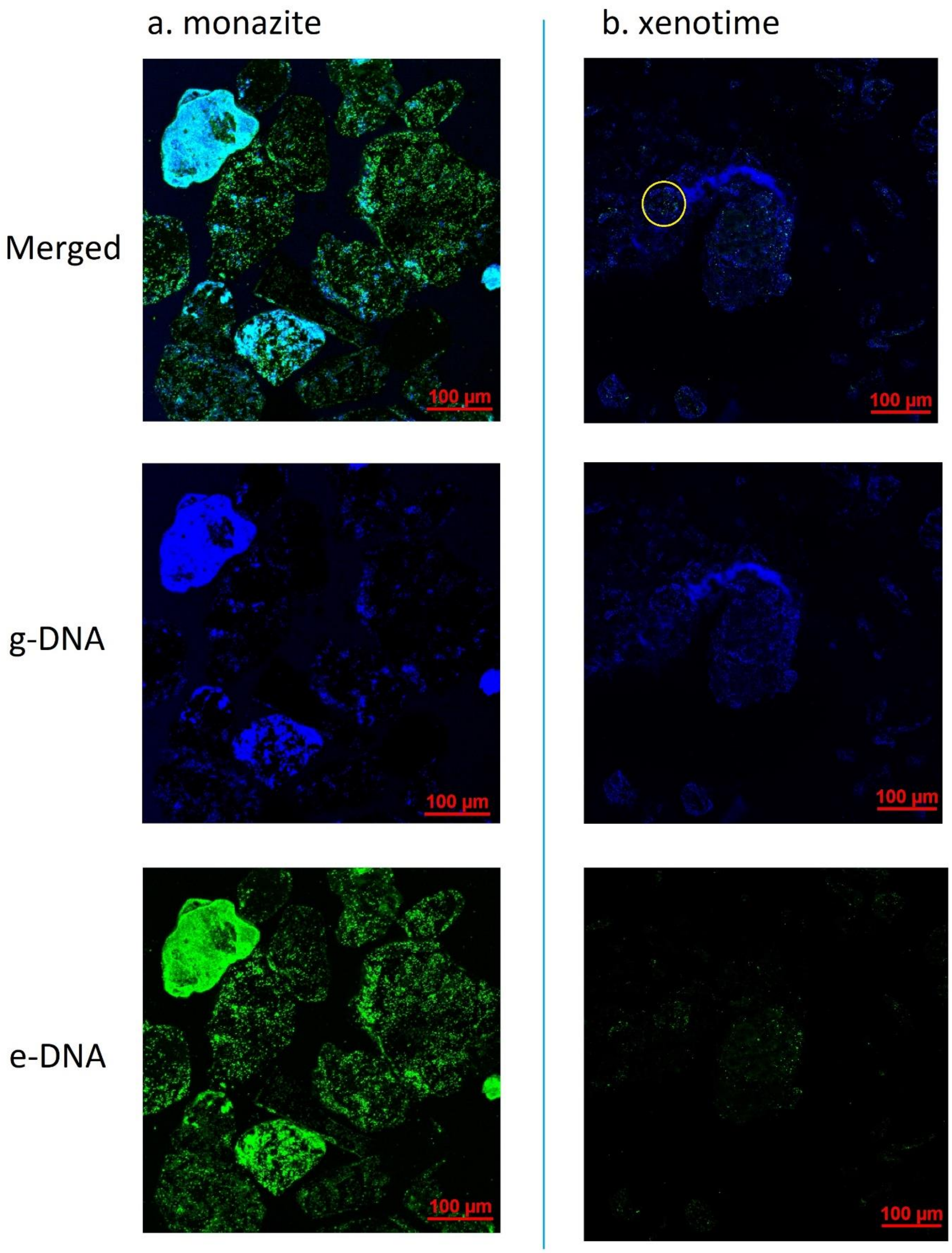

Figure S2. The general pattern of eDNA production on monazite (**a**) and xenotime (**b**) based on confocal laser scanning microscopy (CLSM). The green fluorescent represents eDNA (eDNA) and blue represent genomic DNA (gDNA). More eDNA was produced using monazite. Yellow circle marks the xenotime grain used for 3D re-construction in Figure 5.

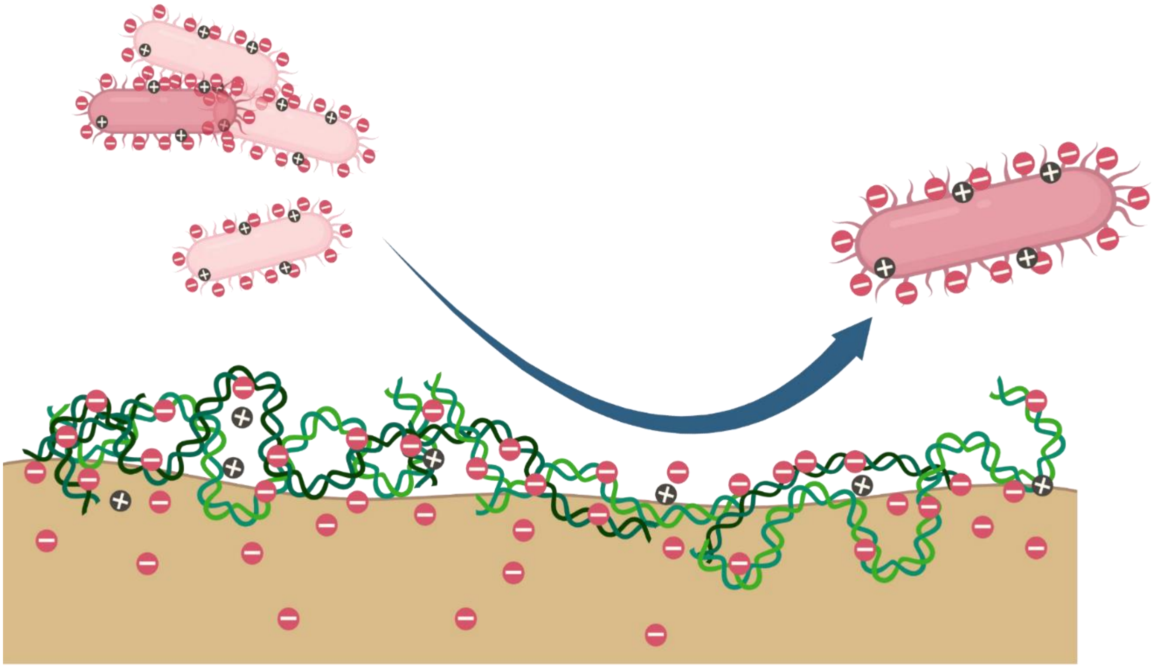

Figure S3. The interaction of *Klebsiella aerogenes* cells with surface pre-conditioned with eDNA.

Statistical data

Raw stats - 1

X	A			B			D		
Time (minutes)	Ctrl 1(P deficient)			Ctrl 2 (+P)			10^7 cell/mL		
X	Mean	SD	N	Mean	SD	N	Mean	SD	N
0.000	100.000	0.000	3	100.000	0.000	3	100.000	0.000	3
1.000	89.600	0.800	3	89.867	1.665	3	92.267	4.406	3
10.000	80.800	6.835	3	82.400	2.884	3	65.333	3.781	3
30.000	47.467	3.781	3	49.333	2.444	3	40.000	4.233	3
60.000	49.867	5.445	3	53.600	2.117	3	33.600	2.117	3
120.000	58.667	3.607	3	59.467	7.259	3	38.400	3.200	3
180.000	58.133	3.946	3	61.333	10.653	3	22.933	3.331	3
240.000	59.200	2.884	3	53.867	2.444	3	26.133	2.013	3

Time (minutes)	10^6 cell/mL			10^7 cell/mL			5 x 10^7 cell/mL		
X	Mean	SD	N	Mean	SD	N	Mean	SD	N
0.000	100.000	0.000	3	100.000	0.000	3	100.000	0.000	3
1.000	89.367	6.369	3	92.267	4.406	3	47.433	7.826	3
10.000	59.667	3.958	3	65.333	3.781	3	57.033	4.620	3
30.000	47.067	1.405	3	40.000	4.233	3	34.833	5.138	3
60.000	45.067	5.613	3	33.600	2.117	3	68.900	2.200	3
120.000	38.933	2.219	3	38.400	3.200	3	72.133	4.735	3
180.000	38.033	1.026	3	22.933	3.331	3	76.233	4.412	3
240.000	37.767	2.294	3	26.133	2.013	3	93.367	11.582	3

Comparison - 1

Dunnett's multiple comparisons test	Summary	Adjusted P Value
Row 1		
10^7 cell/mL vs. Ctrl 1(P deficient)		
10^7 cell/mL vs. Ctrl 2 (+P)		
10^7 cell/mL vs. 10^6 cell/mL		
10^7 cell/mL vs. 5 x 10^7 cell/mL		
Row 2		
10^7 cell/mL vs. Ctrl 1(P deficient)	ns	0.7266
10^7 cell/mL vs. Ctrl 2 (+P)	ns	0.7963
10^7 cell/mL vs. 10^6 cell/mL	ns	0.9055
10^7 cell/mL vs. 5 x 10^7 cell/mL	**	0.0070
Row 3		
10^7 cell/mL vs. Ctrl 1(P deficient)	ns	0.0969
10^7 cell/mL vs. Ctrl 2 (+P)	*	0.0116
10^7 cell/mL vs. 10^6 cell/mL	ns	0.3483
10^7 cell/mL vs. 5 x 10^7 cell/mL	ns	0.1905
Row 4		
10^7 cell/mL vs. Ctrl 1(P deficient)	ns	0.2134
10^7 cell/mL vs. Ctrl 2 (+P)	ns	0.1029
10^7 cell/mL vs. 10^6 cell/mL	ns	0.2011
10^7 cell/mL vs. 5 x 10^7 cell/mL	ns	0.5443

Dunnett's multiple comparisons test	Summary	Adjusted P Value
Row 5		
10^7 cell/mL vs. Ctrl 1(P deficient)	ns	0.0563
10^7 cell/mL vs. Ctrl 2 (+P)	***	0.0009
10^7 cell/mL vs. 10^6 cell/mL	ns	0.1325
10^7 cell/mL vs. 5 x 10^7 cell/mL	***	0.0001
Row 6		
10^7 cell/mL vs. Ctrl 1(P deficient)	**	0.0056
10^7 cell/mL vs. Ctrl 2 (+P)	ns	0.0566
10^7 cell/mL vs. 10^6 cell/mL	ns	0.9968
10^7 cell/mL vs. 5 x 10^7 cell/mL	**	0.0026
Row 7		
10^7 cell/mL vs. Ctrl 1(P deficient)	***	0.0010
10^7 cell/mL vs. Ctrl 2 (+P)	*	0.0409
10^7 cell/mL vs. 10^6 cell/mL	*	0.0245
10^7 cell/mL vs. 5 x 10^7 cell/mL	***	0.0004
Row 8		
10^7 cell/mL vs. Ctrl 1(P deficient)	***	0.0005
10^7 cell/mL vs. Ctrl 2 (+P)	***	0.0004
10^7 cell/mL vs. 10^6 cell/mL	**	0.0080
10^7 cell/mL vs. 5 x 10^7 cell/mL	*	0.0186

Raw stats - 2

Time (minutes)	0.5% Slury			1% slurry			2% Slurry		
X	Mean	SD	N	Mean	SD	N	Mean	SD	N
0.000	100.000	0.000	3	100.000	0.000	3	100.000	0.000	3
1.000	86.000	10.113	3	92.267	4.406	3	55.567	2.550	3
10.000	91.333	5.605	3	65.333	3.781	3	27.800	1.825	3
30.000	63.200	8.551	3	40.000	4.233	3	24.600	0.721	3
60.000	59.133	11.002	3	33.600	2.117	3	17.500	1.967	3
120.000	48.200	10.371	3	38.400	3.200	3	13.000	0.458	3
180.000	60.067	11.705	3	22.933	3.331	3	13.833	2.023	3
240.000	61.933	9.646	3	26.133	2.013	3	12.800	1.833	3

Time (minutes)	5% Slurry			10% Slurry		
X	Mean	SD	N	Mean	SD	N
	100.000	0.000	3	100.000	0.000	3
	38.267	2.811	3	30.300	6.678	3
	20.500	0.458	3	12.200	0.361	3
	13.433	2.695	3	8.767	0.416	3
	10.600	2.623	3	8.267	0.577	3
	8.867	1.401	3	9.100	0.300	3
	11.967	2.250	3	9.067	0.839	3
	11.633	1.710	3	8.733	0.702	3

Comparison - 2

Dunnett's multiple comparisons test	Below threshold?	Summary	Adjusted P Valu
Row 1			
1% slurry vs. Ctrl 1(P deficient)			
1% slurry vs. Ctrl 2 (+P)			
1% slurry vs. 0.5% Slury			
1% slurry vs. 2% Slurry			
1% slurry vs. 5% Slurry			
1% slurry vs. 10% Slurry			
Row 2			
1% slurry vs. Ctrl 1(P deficient)	No	ns	0.7980
1% slurry vs. Ctrl 2 (+P)	No	ns	0.8646
1% slurry vs. 0.5% Slury	No	ns	0.8194
1% slurry vs. 2% Slurry	Yes	**	0.0026
1% slurry vs. 5% Slurry	Yes	***	0.0006
1% slurry vs. 10% Slurry	Yes	**	0.0014
Row 3			
1% slurry vs. Ctrl 1(P deficient)	No	ns	0.1192
1% slurry vs. Ctrl 2 (+P)	Yes	*	0.0147
1% slurry vs. 0.5% Slury	Yes	*	0.0140
1% slurry vs. 2% Slurry	Yes	**	0.0023
1% slurry vs. 5% Slurry	Yes	**	0.0056
1% slurry vs. 10% Slurry	Yes	**	0.0041
Row 4			
1% slurry vs. Ctrl 1(P deficient)	No	ns	0.2619
1% slurry vs. Ctrl 2 (+P)	No	ns	0.1267
1% slurry vs. 0.5% Slury	No	ns	0.0779
1% slurry vs. 2% Slurry	No	ns	0.0581
1% slurry vs. 5% Slurry	Yes	**	0.0056
1% slurry vs. 10% Slurry	Yes	*	0.0151
Row 5			
1% slurry vs. Ctrl 1(P deficient)	No	ns	0.0685
1% slurry vs. Ctrl 2 (+P)	Yes	**	0.0012
1% slurry vs. 0.5% Slury	No	ns	0.1372
1% slurry vs. 2% Slurry	Yes	**	0.0024
1% slurry vs. 5% Slurry	Yes	**	0.0013
1% slurry vs. 10% Slurry	Yes	**	0.0036
Row 6			
1% slurry vs. Ctrl 1(P deficient)	Yes	**	0.0071
1% slurry vs. Ctrl 2 (+P)	No	ns	0.0693
1% slurry vs. 0.5% Slury	No	ns	0.5578
1% slurry vs. 2% Slurry	Yes	*	0.0123
1% slurry vs. 5% Slurry	Yes	**	0.0034
1% slurry vs. 10% Slurry	Yes	**	0.0098
Row 7			
1% slurry vs. Ctrl 1(P deficient)	Yes	**	0.0012
1% slurry vs. Ctrl 2 (+P)	Yes	*	0.0495
1% slurry vs. 0.5% Slury	No	ns	0.0679
1% slurry vs. 2% Slurry	No	ns	0.0721
1% slurry vs. 5% Slurry	Yes	*	0.0413
1% slurry vs. 10% Slurry	Yes	*	0.0396
Row 8			
1% slurry vs. Ctrl 1(P deficient)	Yes	***	0.0006
1% slurry vs. Ctrl 2 (+P)	Yes	***	0.0005
1% slurry vs. 0.5% Slury	No	ns	0.0535
1% slurry vs. 2% Slurry	Yes	**	0.0040
1% slurry vs. 5% Slurry	Yes	**	0.0028
1% slurry vs. 10% Slurry	Yes	**	0.0058
